# Integrating data- and model-driven strategies in systems biology

**DOI:** 10.1186/s12918-018-0562-1

**Published:** 2018-04-24

**Authors:** Yong Wang, Xiang-Sun Zhang, Luonan Chen

**Affiliations:** 1 CEMS, NCMIS, MDIS, Academy of Mathematics and Systems Science, Chinese Academy of Sciences, Beijing, 100190 China; 2 School of Mathematical Sciences, University of Chinese Academy of Sciences, Beijing, 100049 China; 3 Key Laboratory of Systems Biology, SIBS-Novo Nordisk Translational Research Centre for PreDiabetes, Shanghai Institutes for Biological Sciences, Chinese Academy of Sciences, Shanghai, 200031 China; 4 Center for Excellence in Animal Evolution and Genetics, Chinese Academy of Sciences, Kunming, 650223 China

## Abstract

A report of the 11th International Conference on Systems Biology (ISB2017), 18–21 August, Shenzhen, China.

## Background

Recently there is a set of important emerging trends in systems biology study. On the one hand, we keep witnessing the revolution in the generation of high throughput data in almost every aspect of biological and biomedical sciences. In addition to personalized genomics and electronic health records, we also embrace the large scale epigenetics data and image data. The fact is clearly that almost all biologist and clinical doctors rely heavily on the data generation, analysis, and modeling, i.e., extract meaningful knowledge from the vast amount of data. With the success of big data and artificial intelligence in business in every industry, data-driven will be the long-last trend in systems biology. On the other hand, our knowledge on the biological systems is increasingly accumulated in database and literature. Those knowledge are useful as prior information or validation for the data mining. This calls for the integration of data- and model-driven efforts in the experimental design, data generation, data analysis, and model validation. Data-driven has the advantage in generating hypothesis and make prediction in an efficient way and model-driven is strong is reasoning and provides mechanism. Integrating the two strategies will achieve the model-data-model cycle in systems biology.

Along with quantitative need to deal with the big data, this energetic interdisciplinary field has kept attracting excellent scientists and making significant progresses to convert the biological data to fundamental insights in biology and medicine. Our International Conference on Computational Systems Biology (ISB), launched ten years ago [1–5], continues to serve as a high-quality platform and brought many researchers and students to freely exchange ideas. The 11th International Conference on Computational Systems Biology (ISB2017) was successfully organized by Chinese Academy of Sciences (CAS) and Southern University of Science and Technology (SUSTech). We hope that the joint efforts of societies, funding agencies, research institutes, and universities will further push the development of computational methodologies, algorithms, and software in systems biology.

## Meeting report

The 11th International Conference on Computational Systems Biology (ISB 2017), co-organized by Southern University of Science and Technology (SUSTech), Computational Systems Biology Society of ORSC and Systems Biology Division of CSBMB was held in Shenzhen, China, August 18–21, 2017. The conference is supported and sponsored by Academy of Mathematics and Systems Sciences of CAS (AMSS), Shanghai Institutes for Biological Sciences of CAS (SIBS), National Natural Science Foundation of China (NSFC), Functional Genome Informatics and Systems Biology Society of CSCB, Systems Biology Technical Committee of IEEE SMC Society.

ISB2017 attracted great leading scientists working in biology, physics, mathematics and computer Science, optimization, statistics, and many other mathematical methods have been widely used in the field.

Following the successful ISB conferences series since 2007, the purpose of ISB 2017 is to extend the international forum for scientists, researchers, educators, and practitioners to exchange ideas and approaches, to present research findings and state-of-the-art solutions in this interdisciplinary field, including theoretical methodology development and its applications in biosciences and researches on various aspects of computational systems biology.

Ninty-three submissions to ISB2017 cover wide range of computational systems biology. Moreover, the reviewers from the Program Committee of ISB2017 selected 14 papers to be recommended for a special issue in BMC Systems Biology after significant extension of their original submission. Each submission has been peer reviewed and evaluated by three independent reviewers on the quality, originality, soundness, and significance of its contributions. Here we focus on some of the highlights of the meeting by categorizing and briefly introducing these selected papers.

Model-driven studies aims to reveal the mechanism via simplifying or abstracting the real world complexity. In this issue, Wei et al. aimed to identify novel therapeutic signatures in Diamond Black-fan anaemia (DBA) and uncovered their mechanisms to model the gene expression, ontology, pathway, and protein-protein interactions. Li et al. modelled the differential regulation networking to provide useful clues for understanding the common dysfunctional regulation mechanisms of gastric cancer progression and discovering new universal drug targets or biomarkers for gastric cancer. Gao et al. proposed a novel method to identify complexes on PPI networks, based on different co-expression information. Shang et al. modelled a host-microbial covariance network was constructed based on the 16 s rRNA and gene expression data of the samples and aimed to provide an important clue in understanding the regulatory mechanism of microbiota in uterine cervix cancer. Ye et al. built two human PPI networks by using data sets with different confidence levels, studied the network properties of the whole human WD40 protein family systematically, and provided rich knowledge for better understanding WD40 proteins’ roles in organizing the PPI network. Liu et al. modelled a special type of biological systems that can be described using ordinary differential equations or continuous Petri nets (CPNs), and proposed a class of fuzzy continuous Petri nets (FCPNs). Wang et al. performed the analysis of the mathematic model by bifurcation theory and numerical simulations and demonstrated that healthy progenitor cells are bestowed a competitive advantage over leukaemia stem cells.

Data driven studies focus on the methods and algorithms for data analysis and integrations to meet the challenge that data is growing astronomically. In this issue, He et al. proposed an algorithm to find sets of gene knock-downs that induce gene expression changes similar to a drug treatment and applied their approaches to five datasets generated from different cancer cell lines. Zou et al. developed a promoter recognition method called 70ProPred by combining position-specific trinucleotide propensity based on single-stranded characteristic (PSTNP_SS_) with the electron-ion interaction potential values for trinucleotides (PseEIIP) to predict sigma70 promoters in prokaryote. Pai et al. sequenced the transcriptomes of survivin (*birc5*) gene knock-down experimental and wild-type control zebrafish embryos and a differential expression (DE) gene list was obtained for traditional functional enrichment analysis. They demonstrated that incorporating genes near or overlapped with DE lncRNAs into the DE gene list outperformed the traditional enrichment analysis method for effective biological functional interpretations. Gong et al. put forward a novel pancreas segmentation network with multi-layer up-sampling structure for efficient computational recognition and segmentation of cancer target organ from medical images. Lei et al. proposed a new algorithm, improved Flower Pollination algorithm to identify essential proteins by combining network topology with gene expression data, subcellular localization and protein complexes information. Li et al. performed RNA-Seq and characterized the expression profiles of differentiated tissues from *Oryza sativa* Zhonghua 11, including leaves, sheath, stamen, pistil, lemma and palea of the booting stage, and embryo, endosperm, lemma and palea of the mature grain stage and generated tissue-specific models and investigated the shift of metabolic patterns, and the discrepancy between transcriptomic and metabolic level. Cui et al. extended the classical result of the fixation probability of beneficial mutations obtained by Haldane, and estimate the fixation probability of a beneficial mutation with a reduced generation time in a changing environment.
